# Supplementary material for: The interactome of intact mitochondria by cross-linking mass spectrometry provides evidence for coexisting respiratory supercomplexes
Source: Mol Cell Proteomics. 2017 Dec 8;17(2):216–32. doi: 10.1074/mcp.RA117.000470 (PMC5795388; doi:10.1074/mcp.RA117.000470)
Supplement: Supplemental Data [file supp_RA117.000470_133922_0_supp_23978_fzffwf.zip › spectra_annotation/mito_DR_spectra_annotation/213-1-59-1-11-1.pdf]

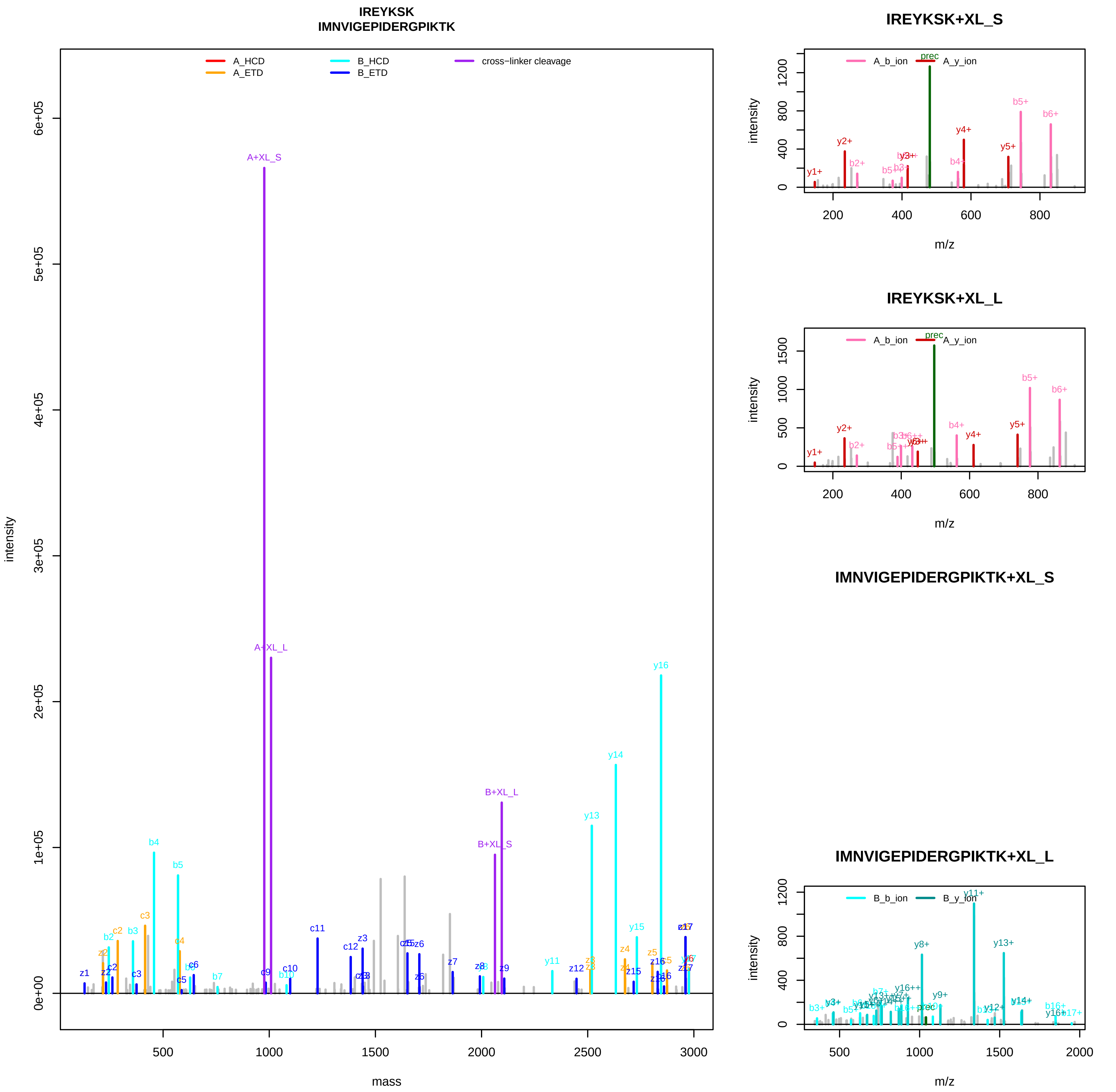

IREYKSK
IMNVIGEPIDERGPIKTK
A_HCD
A_ETD
B_HCD
B_ETD
cross−linker cleavage
intensity
mass
0e+00
1e+05
2e+05
3e+05
4e+05
5e+05
6e+05
500
1000
1500
2000
2500
3000
A+XL_S
A+XL_L
B+XL_L
B+XL_S
y16
y14
y13
y15
y11
b4
b5
c3
c4
b2
b3
c2
z1
z2
c5
c6
b7
c9
c10
b10
c11
c12
z3
z5
z6
z7
z8
z9
z12
z15
z16
c16
z17
c17
IREYKSK+XL_S
IREYKSK+XL_L
IMNVIGEPIDERGPIKTK+XL_S
IMNVIGEPIDERGPIKTK+XL_L
A_b_ion
A_y_ion
B_b_ion
B_y_ion
prec
m/z
y1+
y2+
b2+
y4+
y5+
b4+
b5+
b6+
y8+
y11+
y13+
y9+
y12+
y14+
b16+
y16+
b17+
